# Chemical Constituents and Biological Activities of *Oxandra* (Annonaceae): A Review

**DOI:** 10.1002/cbdv.202503313

**Published:** 2026-01-06

**Authors:** Rayssa Cota Lopes, Francisco Paiva Machado, Mateus de Freitas Brito, Thalisson Amorim de Souza, Lucas Silva Abreu

**Affiliations:** ^1^ Laboratório de Química dos Produtos Naturais Instituto de Química Universidade Federal Fluminense Niterói Brazil; ^2^ Laboratório de Tecnologia de Produtos Naturais Faculdade de Farmácia Universidade Federal Fluminense Niterói Brazil; ^3^ Laboratório Multiusuário de Caracterização e Análises Instituto de Pesquisa em Fármacos e Medicamentos (IpeFarM) Universidade Federal da Paraíba João Pessoa Brazil

**Keywords:** anonnaceae, oxandra, review, biological activity, natural product

## Abstract

*Oxandra* (Annonaceae) comprises 29 species distributed across South and Central America. Despite this diversity, the genus remains under‐investigated. This study presents the first comprehensive review of the chemical composition and biological activities of *Oxandra* species, utilizing databases such as SciFinder, ScienceDirect, PubMed, and Google Scholar, with a period up to 2025. As results, only six species had their chemical and/or biological studies reports. Nevertheless, 64 compounds were identified, including alkaloids, flavonoids, terpenes, and steroids. Reported biological activities include anticorrosive, antioxidant, antimicrobial, cytotoxic, antiparasitic, anti‐inflammatory, and antihyperglycemic effects. Significantly, the compound velutinam demonstrated potent inhibitory activity against DYRK1A (0.6 µM) and CDK1/cyclin B (1.5 µM), kinases associated with neurodegenerative disorders, indicating the way for promising studies in the fields of phytochemistry and pharmacology. This review evaluates the phytochemistry and biological activities of the genus *Oxandra*, integrating chemical and pharmacological knowledge to propose future directions for research on this genus.

## Graphical abstract

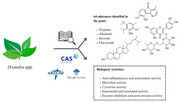

## 1 Introduction

The Annonaceae family is a member of the Magnoliales order with the largest number of known plant species, accounting for 2400 species distributed in around 130 genera [1]. Brazil is home to 29 genera and 386 species of this family, distributed mainly in the Amazon, but also in the Atlantic Forest and Cerrado [2]. The chemical diversity present in *Annonaceae* species covers a wide range of aromatic substances, phenolic acids, tannins, flavonoids, catechins, proanthocyanidins, essential oils, terpenes, steroids, alkaloids, acetogenins, carbohydrates, lipids, proteins, lactones, vitamins, carotenes, saponins, among other compounds [3]. The chemical variety found in members of the Annonaceae family, and their pharmacological properties motivate scientific interest in research into the family.

The genus *Oxandra*, belonging to the Annonaceae family, comprises a total of 29 species distributed throughout northern South America and in several Central American countries. It consists mainly of small to medium‐sized trees, with most species reaching up to 20 m in height, although some species, such as *Oxandra sphaerocarpa* and *Oxandra xylopioides*, exceed 40 m. Their leaves are simple, with short petioles and blades that are generally elliptical, oval, or obovate in shape, with a papery texture. The flowers are small, with loose sepals and six intertwined petals, ranging in color from white to yellow [4, 5].

The fruits are apocarpous, consisting of a free monocarp, which can vary from ovoid to globular, with a single seed with a well‐developed rumen. Although they are edible fruits, they are not as economically significant as *Annona muricata* and *Annona squamosa*. The distribution area of *Oxandra* ranges from Mexico and the Antilles to southeastern Brazil and Bolivia, with the Western Amazon presenting the greatest diversity. Most species are found in humid forests on firm ground, igapós, and floodplains, although some can adapt to more arid environments, such as the cerrado, caatinga, and campinaranas, at altitudes ranging from sea level to 1700 m [5].

The genus has identified oxoaporfine alkaloids, benzylisoquinoline alkaloids, terpenoids, flavonoids, and steroids, among other compounds, isolated mainly from leaves and twigs. The biological activities reported for these extracts include antioxidant, anti‐inflammatory, insecticidal effects, and potential antitumor and antimicrobial activity. To date, there are no reports in the literature of review articles that describe the phytochemical and pharmacological potential of the *Oxandra* genus. Therefore, this article aims to gather and present the isolated substances and bioactive activities described to date in the genus *Oxandra*.

## 2 Methodology

The literature review on the *Oxandra* genus was conducted by searching the CAS SciFinder, Google Scholar, and MEDLINE electronic databases using only the descriptor “*Oxandra*” associated with the Boolean operator “intitle”: to locate only documents whose title and/or abstract directly mention the genus of interest. The period of publication considered in this study was up to 2025. The search was not limited by language and type of study.

**FIGURE 1:**
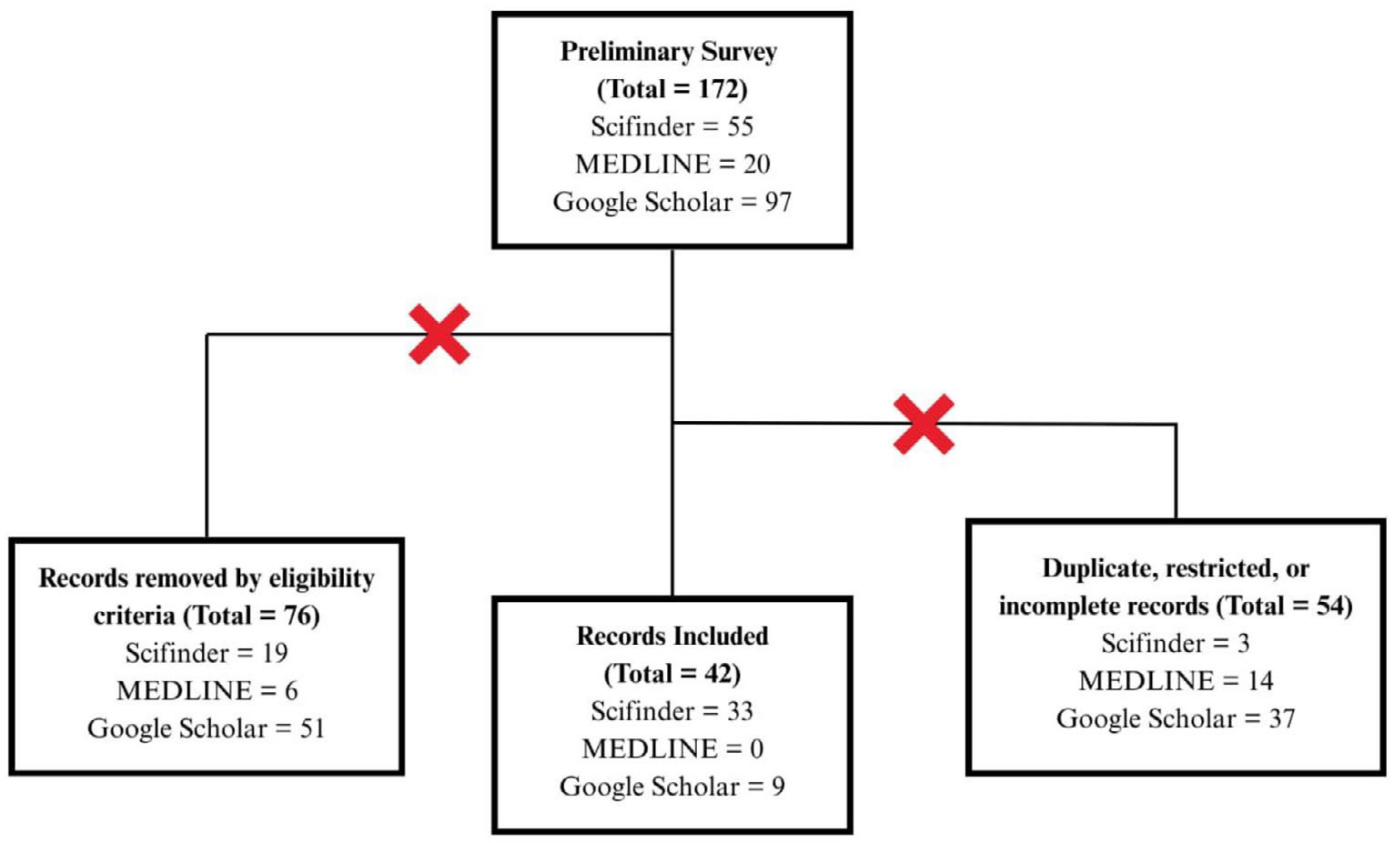
Process used for the exclusion and selection of articles.

The preliminary survey obtained a total of 172 results up to April 2025, with the first publication dating back to 1985. The studies that met the following eligibility criteria were manually selected: Focus primarily on the isolation and/or identification of substances in any species belonging to the genus *Oxandra* or mentioning their biological activities from extracts or isolated compounds. Seventy‐six documents were removed for not meeting the established criteria, and 54 for being duplicated, restricted, or incomplete. In the end, 42 references were used for the composition of this review article. All data presented, including chemical compounds, have been manually validated through cross‐checking sources. Figure 1 summarizes the steps and depicts the criteria applied.

## 3 Results

**FIGURE 2:**
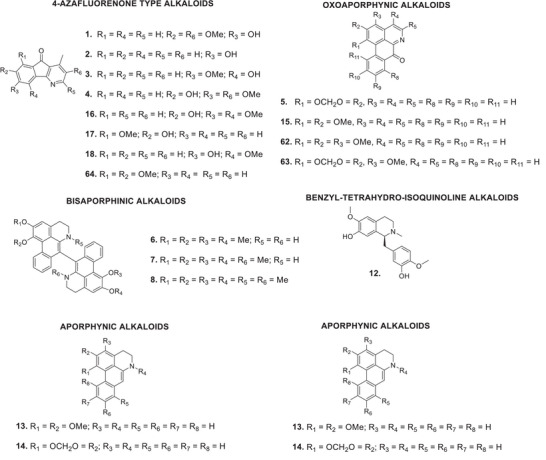
Alkaloids identified in *Oxandra* genus.

**FIGURE 3:**
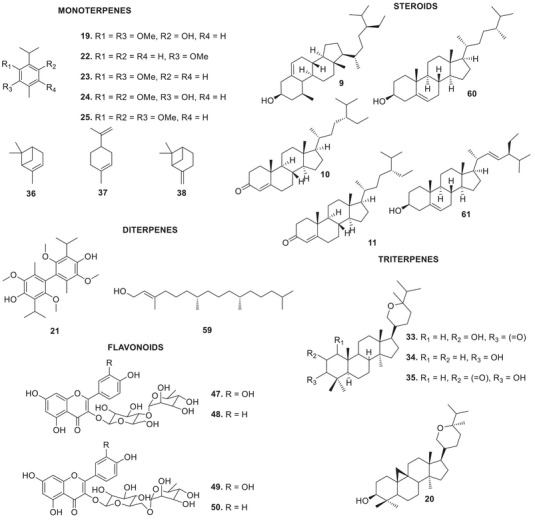
Mono‐, di‐, triterpenoids, steroids, and flavonoids identified in *Oxandra* genus.

**FIGURE 4:**
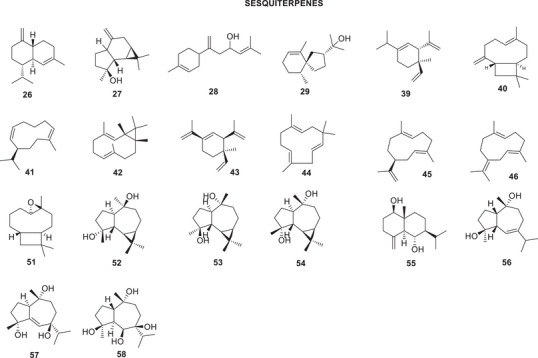
Sesquiterpenoids identified in *Oxandra* genus.

The bibliographic survey identified that, out of a total of 29 taxonomically identified *Oxandra* species, only six species have been chemically and pharmacologically investigated. These are: *O. xylopioides* Diels, *Oxandra espintana* Baill., *Oxandra asbeckii* (Pulle) R.E.Fr., *Oxandra lanceolata* (Sw.) Baill., *Oxandra sessiliflora* R.E.Fr., and *Oxandra longipetala* R.E.Fr. In total, 34 terpenoids, 21 alkaloids, 5 steroids and 4 flavonoids, and several biological activities were reported, as shown in Tables 1 and 2 and Figures 2, 3, 4. The results obtained for each of the species addressed are as follows:

**TABLE 1:** Compounds isolated or identified in species of the genus *Oxandra*.

Species	Plant part	Extract or essential oil	Identified substances	References
*Oxandra xylopioides* Diels	Stem bark	Ethanol	Alkaloids (4‐azafluorenone) 6‐Hydroxy‐2,7‐dimethoxyonychine (**1**) 6‐Hydroxyonychine (**2**) 5‐Hydroxy‐6‐methoxyonychine (**3**) 2,6‐Dimethoxy‐7‐hydroxyonychine (**4**)	[43, 44]
		Chloroform	Oxoaporphine alkaloids Liriodenine (**5**) Alcalóides (4‐azafluorenonas) 5‐Hydroxy‐6‐methoxyonychine (**3**)	[45]
		Petroleum ether	Bisaporphine alkaloids Urabaine (**6**) *N*‐Methylurabaine (**7**) *N*,*N*′‐methylurabaine (**8**) Steroids Sitosterol (**9**) Stigmast‐4‐en‐3‐one (**10**) Sitostenone (**11**)	[6, 7]
		Dichloromethane	Benzil‐tetrahydro‐isoquinoline alkaloids Reticuline (**12**) Aporphine alkaloids Nornuciferine (**13**) Anonaine (**14**) Bisaporphine alkaloids Urabaine (**6**) *N*‐Methylurabaine (**7**) *N*,*N*′‐Methylurabaine (**8**) Oxoaporphine alkaloids Lisicamine (**15**) Liriodenine (**5**) Alkaloids (4‐azafluorenone) 7‐Hydroxy‐5,6‐dimethoxyonychine (**16**) 7‐Hydroxy‐8‐methoxyonychine (**17**) 6‐Hydroxy‐5‐methoxyonychine (**18**)	[6]
	Leaves	Petroleum ether	Monoterpenes Isoespintanol (**19**)	[8, 10, 12, 14, 15, 16, 17, 18, 19, 20, 21, 22, 23, 25, 26, 27, 28, 46, 47]
		Dichloromethane	Triterpenes Berenjenol (**20**) Diterpenes Diisoespintanol (**21**)	[8, 9, 11, 13, 24]
*Oxandra espintana* Baill.	Stem bark	Petroleum ether	Monoterpenes *o‐*Methylcarvacrol (**22**) Thymoquinol dimethyl ether (**23**) Espintanol (**24**) *o*‐Methylespintanol (**25**)	[29]
	Leaves	Essential oil	Sesquiterpenes γ‐Cadinene (**26**) Espatulenol (**27**) β‐Atlantol (**28**) Hinesol (**29**)	[30]
*Oxandra asbeckii* (Pulle) R.E.Fr.	Stem bark	Ethyl acetate	Aristolactams alkaloids Aristolactam AII (**30**) Aristolactam BII (**31**) Velutinam (**32**)	[33]
	Leaves	Dichloromethane	Oxoaporphine alkaloids Liriodenine (**5**)	[31]
		Hexane	Sesquiterpenes Espatulenol (**27**) Triterpenes 2,3‐Dioxo‐oxandrane (**33**) 3‐Hydroxy‐oxandrane (**34**) 3‐Hydroxy‐oxandran‐2‐one (**35**)	[31, 32]
		Total alkaloid	—	[34]
*Oxandra lanceolata* (Sw.) Baill.	Leaves	Essential oil	Sesquiterpenes Espatulenol (**27**) Monoterpenes α‐Pinene (**36**) Limonene (**37**) β‐Pinene (**38**)	[35]
*Oxandra sessiliflora* R.E.Fr.	Leaves	Essential oil	Sesquiterpenes δ‐Elemene (**39**) β‐Cariofilene (**40**) Germacrene A, B, and D (**45**, **46**, and **41**) Bicyclogermacrene (**42**) α‐Pinene (**36**) β‐Pinene (**38**) β‐Elemene (**43**) α‐Humulene (**44**) Espatulenol (**27**)	[36]
		Ethyl acetate	Flavonoids Quercetine‐3‐O‐α‐l‐rhamnopyranosyl‐(1→4)‐β‐d‐glucopyranoside (**47**) Canferol‐3‐O‐α‐l‐rhamnopyranosyl‐(1→4)‐β‐d‐glucopyranoside (**48**) Rutine (**49**) Canferol‐3‐O‐rutinoside (**50**)	[37]
		Hexane	Sesquiterpenes Caryophyllene oxide (**51**) Espatulenol (**27**)	[38]
		Chloroform	Sesquiterpenes 4α,10β‐Aromadendranediol (**52**) 4β,10α‐Aromadendranediol (**53**) 4α,10α‐Aromadendranediol (**54**) 1β,6α‐Dihydroxyeudesm‐4(15)‐ene (**55**) 4β,10α‐Dihydroxy‐guai‐6‐ene (**56**) 4α,7β,10α‐Trihydroxyguai‐5‐ene (**57**) 4β,6β,7β,10α‐Tetrahydroxy‐guaiane (**58**) (*E*)‐fitol (**59**) Steroids Campesterol (**60**) Sitosterol (**9**) Stigmasterol (**61**)	[37, 39]
*Oxandra longipetala* R.E.Fr.	Leaves	Ethanol (alkaloid fraction)	Oxoaporphine alkaloids Lisicamine (**15**) *o*‐Methylmoschatoline (**62**) Atherospermidine (**63**) Liriodenine (**5**) Aporphine alkaloids Nornuciferine (**13**) Anonaine (**14**)	[41]
	Stem bark	Ethanol	Oxoaporphine Alkaloids Lisicamine (**15**) *o*‐Methylmoschatoline (**62**) Liriodenine (**5**) Alkaloids (4‐azafluorenone) 6‐Hydroxy‐5‐methoxyonychine (**18**) 5‐Hydroxy‐6‐methoxyonychine (**3**) *o‐*Methylmacondine (**64**) 7‐Hydroxy‐8‐methoxyonychine (**17**)	[40, 42]

**TABLE 2:** Relation between biological activity and the classes of compound obtained from *Oxandra* spp.

Class	Subclass	Biological activity	References
Terpenes	Monoterpenes	Anti‐inflammatory; antioxidant activity; antispasmodic; cardioprotective; antibacterial and antibiofilm potential against *Pseudomonas aeruginosa*; antifungal potential against *Candida* spp.; preventive effect on the development of early‐stage diabetes; anti‐leishmania; anti‐*Trypanosoma* activity	[8, 10, 12, 14, 15, 16, 17, 18, 19, 20, 21, 22, 23, 25, 26, 27, 28, 29, 46, 47]
	Sesquiterpenes	Acaricidal activity against females of *Tetranychus urticae*; cytotoxic activity against tumor cells (HL‐60)	[30, 36, 39]
	Diterpenes	Antifungal potential against *Candida* spp.	[24]
	Triterpenes	Anti‐inflammatory and insecticidal activity against larvae of *Spodoptera frugiperda*	[8, 9, 11, 13]
Alkaloids	Aristolactams alkaloids	Inhibition of DYRK1A (IC_50_ = 0.6 µM) and CDK1/cyclin B (CI_50_ = 1.5 µM)	[33]
	Total alkaloid	Corrosion inhibition action on C38 steel	[34]

### 3.1 *O. xylopioides* Diels

The species *O. xylopioides* Diels, scientifically synonymous with *Oxandra major* R.E.Fr., is popularly known in Brazil as “Envira‐preta,” “Envira‐vassourinha,” “Inviera,” or “Invira‐preta.” Trees of this species can reach between 3 and 45 m in height, with oblong to ovate leaves and a warty texture. The inflorescences have one to seven flowers, with yellow, cream, or white petals. The fruits are monocarpic, obovoid to ellipsoid in shape, varying in color from green to red during ripening. The flowers and fruits of the species have a strong, pleasant odor. The seeds are ellipsoid, measuring 10–18 mm, with a dotted surface [5].

The species is found in the North (Acre, Amazonas, Pará, and Rondônia) and Central‐West (Mato Grosso) regions of Brazil, as well as in regions of neighboring countries such as Bolivia, Peru, Ecuador, Colombia, Venezuela, and French Guiana. In most of the places where the species occurs, its wood is used in construction. In addition, steam baths made from the leaves are used to treat fever in Ecuador. Currently, it is the most researched species of the *Oxandra* genus in terms of its chemical composition [5].

Several alkaloids were identified in the extract of the stem bark of *O. xylopioides*. From the alkaloid fraction of the ethanolic extract, 6‐hydroxy‐2,7‐dimethoxyonychine (or oxylopidine) (**1**), 6‐hydroxyonychine (or oxylopinine) (**2**), 5‐hydroxy‐6‐methoxyonychine (or oxylopine/isoursuline) (**3**), and 2,6‐dimethoxy‐7‐hydroxyonychine (**4**). From the chloroform fraction of the ethanolic extract, the alkaloids liriodenine (**5**) and 5‐hydroxy‐6‐methoxyonychine (**3**) were isolated [43, 44, 45]. From the petroleum ether extract, the bisaporphins urabain (**6**), *N*‐methylurabain (**7**), and *N*,*N*′‐dimethylurabain (**8**) were obtained, in addition to the steroids sitosterol (**9**), stigmast‐4‐en‐3‐one (**10**), and sitostenone (**11**) [6, 7].

The following alkaloids were isolated from the basic fraction of the dichloromethane extract: reticuline (**12**), nornuciferine (**13**), anonine (**14**), lysicamine (**15**), the aforementioned liriodenine (**7**), 7‐hydroxy‐5,6‐dimethoxyonychine (darienine) (**16**), 7‐hydroxy‐8‐methoxyonychine (macondine) (**17**), and 6‐hydroxy‐5‐methoxyonychine (or ursuline) (**18**) [6, 7].

Other studies have investigated the phytochemistry of the leaf extract of this species. The monoterpene isoespintanol (**19**) was obtained from the petroleum ether extract. From the dichloromethane extract, the cycloartan triterpene berenjenol (**20**) and diisoespintanol (**21**) were isolated [8, 9, 10, 11, 12]. Among the substances reported, berenjenol (**20**) and isoespintanol (**19**) were the main targets of investigations into their bioactive properties:

#### 3.1.1 Anti‐Inflammatory Activity

Berenjenol (**20**) and isoespintanol (**19**) were evaluated for their anti‐inflammatory activity. The compounds were tested against edema induced by carrageenan injection into the rat paw. The application of berenjenol (**20**) resulted in a clear reduction in edema of 44%, 64%, and 51% at 1, 3, and 5 h, respectively. Isoespintanol (**19**), on the other hand, showed a significant reduction in edema only after 3 h, with a 43% decrease in inflammation [8].

Subsequently, another study on the anti‐inflammatory activity of berenjenol (**20**) verified its effectiveness in reducing inflammation of acute and subchronic edema, both caused by the application of TPA (tetradecanoic acid) to rat ears. The results indicate no inhibition of interleukin‐1β (IL‐1β) and tumor necrosis factor‐α (TNF‐α) cytokines but point to significant anti‐inflammatory activity associated with the inhibition of cyclooxygenase‐2 (COX‐2) and inducible nitric oxide synthase (iNOS) enzymes [13].

#### 3.1.2 Insecticide Activity

The insecticidal activity of berenjenol (**20**) was also investigated. The compound was applied at concentrations of 25, 50, 100, 200, and 400 ppm in an artificial diet to second instar *Spodoptera frugiperda* larvae. A 50% mortality rate was observed in the population exposed to the maximum dose, with LD_50_ and LD_90_ values of 319.6 (245.32–477.68) ppm and 702.03 (523.97–1179.94) ppm, respectively, after 24 h [9].

#### 3.1.3 Antioxidant Activity

Isoespintanol (**19**) showed significant activity in a comparative study with thymol and butylated hydroxytoluene (BHT), commercial antioxidants widely used. In a test using the ferric reducing antioxidant power (FRAP) method, the electron transfer capacity of isoespintanol (**19**) was evaluated as more effective than that of both competitors [14, 15].

The ABTS (2,2′‐azino‐bis (3‐ethylbenzothiazoline‐6‐sulfonic acid) and DPPH (2,2‐diphenyl‐1‐picrylhydrazyl) assays performed in comparison with BHT suggest that the commercial antioxidant has a greater capacity to scavenge free radicals through proton donation [14]. The result of the DPPH test compared with thymol indicated isoespintanol (**19**) as the most active [15].

Subsequent studies on the effects of adding isoespintanol (**19**) on the oxidative stability of palm olein, a food product used in frying, show a 39% reduction in hydroperoxide production under accelerated oxidation conditions. The effectiveness of isoespintanol (**19**) is greater than that of BHT, which achieved only a 10% reduction and showed lower thermal resistance. The evaluation of the cytotoxic activity of isoespintanol (**19**) at 100 µM in murine macrophages established the possible viability of its application as a food additive [16].

Finally, another assessment of isoespintanol (**19**) as a reactive oxygen species (ROS) scavenger, carried out using the oxygen radical absorbance capacity (ORAC) methodology, points to a lower antioxidant capacity than butylated hydroxyanisole (BHA), a commercial synthetic antioxidant. However, isoespintanol (**19**) showed greater reactivity in neutralizing free radicals compared to BHA. The same study proves the absence of a clastogenic effect of isoespintanol (**19**) on human lymphocyte DNA using the comet assay method [17].

#### 3.1.4 Pharmacological Activities

Other publications focus on the pharmacological activities of isoespintanol (**19**): A preclinical study in rats aimed to evaluate the antispasmodic effect of the compound on the smooth muscles of the intestine, urinary tract, and uterus. The results obtained indicate that isoespintanol (**19**) was able to inhibit contractions induced in tissues by spasmodic compounds by blocking the influx of Ca^2+^ ions [18, 19].

The effects of isoespintanol (**19**) on changes in post‐ischemic cardiac tissue in rats were also determined. The results demonstrate the cardioprotective properties of the compound. Isoespintanol (**19**) was able to limit cell death, reducing the infarct area, as well as decreasing oxidative stress and post‐ischemic dysfunction when administered at the onset of blood flow reperfusion [20].

In addition, isoespintanol (**19**) showed antibacterial potential against *Escherichia coli*, *Pseudomonas aeruginosa*, *Klebsiella pneumoniae*, *Acinetobacter baumannii*, *Proteus mirabilis*, *Staphylococcus epidermidis*, *Staphylococcus aureus*, *Enterococcus faecium*, *Enterococcus faecalis*, *Stenotrophomonas maltophilia*, *Citrobacter koseri*, *Serratia marcescens*, *Aeromonas hydrophila*, and *Providencia rettgeri*. The MIC_90_ values (minimum inhibitory concentration) evaluated by the broth microdilution assays in 96‐well microliter plates ranged from 694.3 to 916.5 µg/mL. While the MIC_50_ ranged from 154.2 to 457.3 µg/mL, the *S. epidermidis* was the most susceptible strain evaluated. In addition, isoespintanol (**19**) showed antibiofilm properties against *P. aeruginosa* mature biofilms, exceeding the efficacy of ciprofloxacin [21].

In terms of antifungal potential, isoespintanol (**19**) and diisospintanol (**21**) have stood out in recent studies as potential adjuvants in the treatment of *Candida* spp. infections, with MIC_90_ between 450.4 and 503.3 µg/mL and 296.7 and 890.3 µg/mL, respectively. Isoespintanol also obtained MIC_90_ values between 326.6 and 500 µg/mL against *Candida tropicalis*. The antifungal action of the compound is associated with its ability to damage the integrity of the plasma membrane and induce ROS, culminating in cell death. Furthermore, the antibiofilm activity of the compound was superior to that of amphotericin B (AFB), a widely used antifungal drug. Consequently, it can be hypothesized that isoespintanol (**19**) may be a more efficacious treatment for fungal infections than for bacterial infections [21, 22, 23, 24, 25, 26].

There are also indications that isoespintanol (**19**) can prevent diabetes in its early stages. The effects recorded against changes induced in rodents with a fructose‐rich diet include normalization of triglycerides, HDL‐cholesterol levels, and insulin resistance index (IRX), as well as a reduction in hepatic glycogen accumulation, inflammatory markers, and oxidative stress [27, 28].

### 3.2 *O. espintana* Baill

The species *O. espintana* Baill., scientifically synonymous with *Oxandra aromatica* Triana & Planch or *Oxandra nitida* R.E.Fr., is popularly known in Brazil as “Araticum‐do‐mato,” “Chaporoasca,” “Envira‐ferro,” “Envireira‐caniceira,” “Imbiú‐amarelo,” and “Pindaíba‐ferreira” [5].

The species can appear as either a tree or a shrub, reaching heights of between 2 and 30 m. Its leaves are ovate to obovate, with a smooth or slightly warty surface and shiny upper side. The inflorescences have one to three flowers, with white or yellow petals and a strong sweet odor. The fruits are ellipsoid to globose monocarps, ranging from green to dark red or black when ripe. The seeds are ellipsoid with a striated and ruminated surface [5].

The geographical distribution of the species covers states in the southeast (Rio de Janeiro, Espírito Santo, and Minas Gerais), northeast (Bahia), and north (Amazonas and Acre) of Brazil. It also includes regions in Venezuela, Colombia, Peru, and Bolivia. In each of these regions, the traditional uses of the plant vary: In Peru, the bark is used to treat rheumatism; The fruit is eaten as food in Bolivia and Colombia; And the wood is widely used for construction in most regions where the species occurs [5].

Four aromatic monoterpenes were isolated from the petroleum ether extract of the stem bark of *O. espintana*: *o*‐methyl carvacrol (**22**) and thymoquinol dimethyl ether (**23**), espintanol (**24**), and *o*‐methylespintanol (**25**) [29]. Analysis of the essential oil from the leaves of the species revealed a predominant composition of sesquiterpenes, with γ‐cadinene (**26**), spathulenol (**27**), β‐atlantol (**28**), and hinesol (**29**) being the major constituents [30].

#### 3.2.1 Antiparasitic Activity

Espintanol (**24**) was subjected to *in vitro* biological assays in 12 strains of *Leishmania* spp. promastigotes and 20 strains of *Trypanosoma cruzi* epimastigotes, demonstrating antiparasitic activity against both. In the first test, the 90% inhibitory concentration (IC_90_) value obtained for espintanol (**24**) (10–50 µg) was intermediate between the two reference leishmanicidal drugs, meglumine antimoniate (>100 µg) and pentamidine (1–5 µg). In the second test, the compound was active in vitro as the two reference products, benznidazole and nifurtimox [29].

#### 3.2.2 Pesticide Activity

The acaricidal activity of *O. espintana* essential oil was evaluated on adult females of the species *Tetranychus urticae*, obtaining significant LC (lethal concentration) of LC_50_ = 13.24 (12.34–14.19) µL/mL of air and LC_90_ = 40.68 (36.10–46.94) µL/mL of air. In addition to reducing fertility and repelling the pest to leaves that had not been treated with the oil [30].

### 3.3 *O. asbeckii* (Pulle) R.E.Fr

The species *O. asbeckii* (Pulle) R.E.Fr. is endemic to the Amazon rainforest, with records of occurrence in Brazil (Acre, Amazonas), Colombia, Venezuela, Suriname, Guyana, and French Guiana, in the regions where the biome extends. It has no Brazilian vernacular name documented in the literature, but is known in other countries as: “Cajao‐dujeco,” “Carguero de vara” (Colombia); “Aso mato,” “Bi pao,” “Gie pawoe,” “Mi‐Pente,” “Moemba,” “Mouamba,” “Bamba Mwemba,” “Npomba” (French Guiana); “Karashiri, Karishiri” (Guyana); “Echte pikapika,” “Foedi‐da,” “Hansoe matoe,” and “Mamba” (Suriname) [5].

The species is a tree or shrub between 4‐ and 25‐m tall. Young branches have dense hair covering that fade over time. The leaf blade is elliptical to narrowly elliptical, with a shiny grayish‐green surface on the upper side. On the underside, there is a grayish to brown coloration and a papery, warty texture. The flowers are solitary, with white, ovate petals. The fruits are clusters of one to seven green monocarps, which ripen purple or black. The seed is ellipsoidal with a slightly dotted to striated surface [5].

There are records of the use of *O. asbeckii* wood for construction in traditional communities in the Amazon region. The material is used in the manufacture of pillars for “carbets” and “takari,” traditional house structures typical of indigenous communities in the Guianas region [5].

The ethyl acetate extract from the stem bark of *O. asbeckii* led to the isolation of three alkaloids, identified as aristolactams AII (**30**), BII (**31**), and velutinam (**32**). This is the first record of aristolactams in the *Oxandra* genus. The alkaloid liriodenine (**5**) was extracted from the dichloromethane extract of *O. asbeckii* leaves. From the hexane extract, the sesquiterpene spathulenol and three methyltriterpenes derived from the same carbon skeleton, oxandrane, were obtained: 2,3‐dioxo‐oxandrane (**33**); 3‐hydroxy‐oxandrane (**34**); and 3‐hydroxy‐oxandran‐2‐one (**35**) [31, 32, 33].

#### 3.3.1 Anticorrosive Activity

The alkaloid extract from *O. asbeckii* leaves was evaluated as an effective corrosion inhibitor in a test with C38 steel exposed to a 1 M hydrochloric acid solution. The study was able to demonstrate that the extract has cathodic and anodic inhibition effects, classified as a mixed‐type inhibitor. It also found that the inhibitory effect is consistent under different thermal conditions and that the adsorption of the extract on the steel surface follows the Langmuir adsorption model [34].

#### 3.3.2 Pharmacological Activity

Aristolactams AII (**30**), BII (**31**), and velutinam (**32**) were evaluated for their ability to inhibit DYRK1A and CDK1/cyclin B kinases, that are related with neurodegenerative disorders, like Alzheimer's disease. While aristolactams AII (**30**) and BII (**31**) showed no significant activity, velutinam (**32**) showed strong inhibitory activity, with IC_50_ values for DYRK1A and CDK1/cyclin B of 0.6 and 1.5 µM, respectively [33].

### 3.4 *O. lanceolata* (Sw.) Baill

The species *O. lanceolata* (Sw.) Baill., scientifically synonymous with *Oxandra virgata* A. Rich., occurs naturally in the following Central American countries: Mexico, Cuba, Jamaica, Haiti, the Dominican Republic, and Puerto Rico. There are also records of the species occurring in Colombia. *O. lanceolata* is known by different vernacular names in the countries where it occurs: Lancewood and Yaya in Cuba; Yaya and Yaya fina in the Dominican Republic; Bois de lance in Haiti; Black lancewood and Lancewood in Jamaica; and Chilcahuite in Mexico [5].

The species is a tree or shrub between 2‐ and 20‐m tall. Young branches are densely covered with hairs, which disappear over time. The leaf blade is rhomboid, ovate to narrowly ovate, grayish‐green in color, opaque, and papery in texture. The flowers appear singly, with white petals and 10 to 15 stamens. The fruits are clusters of 1 to 4 green monocarps, which darken as they ripen. The seed is ellipsoid in shape with transverse striations on its surface. In Mexico, its wood is used for roof construction [5].

A study on the chemical composition of the essential oil from *O. lanceolata* leaves identified 90 compounds, corresponding to 80% of the oil's total composition. The main components are spathulenol (**27**) (13.9%), α‐pinene (**36**) (7.6%), limonene (**37**) (6.6%), and β‐pinene (**38**) (5.6%) [35]. To date, there are no reports in the literature regarding the biological potential of the aforementioned *O. lanceolata*.

### 3.5 *O. sessiliflora* R.E.Fr

The species *O. sessiliflora* is endemic to Brazil, with records of occurrence in Bahia, Ceará, Maranhão, Pará, Paraíba, Piauí, Rio de Janeiro, and Tocantins. It is found in dry vegetation areas, such as caatinga and cerrado biomes, and occasionally in solid ground forests and on sandy soil. It has no registered scientific synonym, and its vernacular name is “Cundurú” [5].

The species is a tree or shrub between 1 and 25 m tall. Young branches are densely covered with hairs, which fade over time. The leaves have a narrow, ovate to obovate blade and a shiny brownish‐green surface on the upper side. The underside of the blade is brown and has sparse to dense hairs. The inflorescences have one to two flowers, with globular buds and ovate petals ranging from white to pink in color, with indumentum on both sides. The fruits are clusters of one to seven ellipsoid monocarps sparsely covered with hairs. They ripen from green to reddish‐black. The seeds are ellipsoid to obovoid, light reddish‐brown in color, and transversely striated on the surface. The species is easy to identify within the *Oxandra* genus due to its small, shiny leaves with sharp tips, sessile flowers with an intense citrus fragrance, and wood that secretes red sap. The plant's application is limited to the use of wood in civil construction [5].

The chemical composition of the essential oil from *O. sessiliflora* leaves includes 33 identified compounds, which correspond to 90% of the total identified volatiles. Chemical identification was performed on four samples of the species, collected at different times of the year, to analyze the intraspecific variation in its composition. The main compounds found are the sesquiterpene hydrocarbons δ‐elemene (**39**) (7.42%–10.59%); β‐caryophyllene (**40**) (5.83%–9.81%); germacrene D (**41**) (17.12%–32.20%); and bicyclogermacrene (**42**) (7.95%–18.91%). In addition to these, only α‐pinene (**36**) and β‐pinene (**38**), β‐elemene (**43**), α‐humulene (**44**), germacrenes A (**45**) and B (**46**), and spathulenol (**27**) were identified in the four oils analyzed [36].

From the ethyl acetate fraction of the ethanolic extract of *O. sessiliflora* leaves, the following flavonoids were isolated: quercetin‐3‐O‐α‐l‐rhamnopyranosyl‐(1→4)‐β‐d‐glucopyranoside (**47**), kaempferol‐3‐O‐α‐l‐rhamnopyranosyl‐(1→4)‐β‐d‐glucopyranoside (**48**), rutin (**49**), and kaempferol‐3‐O‐rutinoside (**50**) [37].

From the hexane fraction of the ethanolic extract of *O. sessiliflora* leaves, the sesquiterpenes caryophyllene oxide (**51**) and spathulenol (**27**) were obtained. From the chloroform fraction, the sesquiterpenes 4α,10β‐aromadendranodiol (**52**), 4β,10α‐aromadendranodiol (**53**), 4α,10α‐aromadendranodiol (**54**), 1β,6α‐dihydroxyeudesm‐4(15)‐ene (**55**), 4β,10α‐dihydroxy‐guaia‐6‐ene (**56**), 4α,7β,10α‐trihydroxy‐guaia‐5‐ene (**57**) and 4β,6β,7β,10α‐tetrahydroxy‐guaiano (**58**), the diterpene (*E*)‐phytol (**59**) and a mixture of three steroids: campesterol (**60**), sitosterol (**9**), and stigmasterol (**61**) [38].

#### 3.5.1 Pharmacological Activities

The essential oil from *O. sessiliflora* leaves showed significant cytotoxic activity when evaluated against five different tumor cell lines: murine (B16F10‐Nex2) and human melanoma (A2058), breast adenocarcinoma (MCF7), leukemia (HL‐60), and cervical carcinoma (HeLa). Human leukemia cells (HL‐60) were the most sensitive to both oils, with similar IC_50_ values of approximately 6 µg/mL. In contrast, B16F10‐Nex2, MCF7, and HeLa cells exhibited moderate sensitivity, with IC_50_ values ranging from 46.5 ± 2.3 to 67.5 ± 3.4 µg/mL. Human melanoma A2058 cells were less sensitive, showing an IC_50_ of 79.8 ± 4.1 µg/mL [36].

Sesquiterpenes **52**–**56** and **58** exhibit antiparasitic activity against *Trypanosoma cruzi* trypomastigotes, with EC_50_ values (effective concentrations capable of inhibiting 50% of *T. cruzi*) ranging from 16.3 to 47.5 µM (positive control benznidazole ranging from 4.2 to 12.3 µM). It was also found that none of the compounds exhibited cytotoxicity against mammalian cells NCTC cells‐clone L929, even at the highest concentration tested (200 µM) [39].

### 3.6 *O. longipetala* R.E.Fr

The species *O. longipetala* R.E.Fr. is found in Central and South America, distributed across the following countries: Nicaragua, Costa Rica, Panama, Colombia, Peru, and Brazil. It is characteristic of tropical forests, especially pre‐montane, deciduous, or gallery forests. These are shrubs or trees 2–10 m tall, with grayish‐green or brownish leaves that are ovate to obovate in shape and non‐verrucose in texture. The surface of the leaf blade is hairless or sparsely hairy. The flowers occur singly, which is an important factor in identifying the species, as they have exceptionally long petals for the *Oxandra* genus. The fruits occur in clusters of 1–10 small, oval‐shaped monocarpels that ripen from green to red or dark purple. The fruits carry one seed each, which is ellipsoid in shape and has a striated texture [5].

The ethanolic extract of *O. longipetala* leaves yielded a fraction of total alkaloids from which four oxoaporphines were isolated and identified: lisicamine (**15**), *o*‐methylmoschatoline (**62**), atherospermidine (**63**), and liriodenine (**7**); and two aporphines, nornuciferine (**13**) and anonain (**14**). The ethanolic extract from the stem bark of the species revealed an alkaloid profile complementary to the above, with four azafluorenones, 6‐hydroxy‐5‐methoxyonichine (**20**), 5‐hydroxy‐6‐methoxyonichine (**3**), *o*‐methylmacondine (**64**), and 7‐hydroxy‐8‐methoxyonichine (**17**), and three oxoaporphines previously identified in the leaves, lisicamine (**15**), *o*‐methylmoschatoline, and liriodenine (**7**) [40, 41].

#### 3.6.1 Pharmacological Activities

The aqueous, dichloromethane, and ethyl acetate extracts from the leaves and stem bark of *O. longipetala* were investigated for their antibacterial activity against seven strains of clinically relevant bacteria: *S. aureus* (ATCC 43300), *S. aureus* (ATCC 29213), *S. aureus* (ATCC 25923), *E. coli* (ATCC 25922), *E. faecalis* (ATCC 29212), *E. faecalis* (ATCC 700603), and *P. aeruginosa* (ATCC 27853). Among the tested extracts, only the dichloromethane fraction showed no significant inhibitory activity. In contrast, the aqueous and ethyl acetate extracts from the stem bark demonstrated the greatest inhibitory effects [42].

The aqueous bark extract produced inhibition zones up to 25 mm against *S. aureus* ATCC 29213 and *E. faecalis* ATCC 51299 at 5000 ppm, the latter showing inhibition comparable to the positive control (chloramphenicol). The ethyl acetate bark extract also showed strong activity, with inhibition zones of 24 mm for *S. aureus* ATCC 29213 and 23 mm for *E. faecalis* ATCC 51299. Other strains, including *E. coli* ATCC 25922 (halo of 13 mm) and *P. aeruginosa* ATCC 27853 (halo of 10 mm), were moderately inhibited at the highest concentrations tested. These findings highlight *E. faecalis* ATCC 51299 and *S. aureus* ATCC 29213 as the most susceptible strains, particularly to the aqueous and ethyl acetate stem bark extracts of *O. longipetala* [42].

## 4 Discussion

**FIGURE 5:**
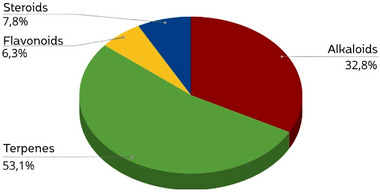
Main classes of compounds identified in in *Oxandra* genus.

Among the classes of chemical compounds found in the *Oxandra* genus described in this literature review, 53.1% of these molecules belong to the terpene class, followed by alkaloids, which represent 32.8% of these substances. Flavonoids and steroids together represent 14.1% of the other classes that can also be found (Figure 5). Considering that phytochemical and biological studies have covered only six of the 29 recognized species of the genus *Oxandra*, this results shows that the genus is still under‐explored but has great chemical potential for prospecting terpenes and alkaloids. The disparity in the coverage of studies evidences the demand for broader research, since the current bias might stem from geographical accessibility, especially for Amazonian species like *O. xylopioides* and *O. asbeckii*, or from well‐established pharmacological interest concerning the Annonaceae family, which is renowned for its wide variety of bioactive compounds [3]. Although ethnobotanical and ethnopharmacological records for *Oxandra* are rare and mostly non‐medicinal, like the use of wood, the historical therapeutic applications of this family could at least spur further research to better link the traditional knowledge with modern phytochemistry.

It was also found that among the classes of substances found in the *Oxandra* genus, steroids and flavonoids were not used to perform biological activity tests. Although there are reports of the isolation of several subclasses of alkaloids, such as oxoaporphine, aporphine, 4‐Azafluorenone, bisaporphine, and benzil‐tetrahydro‐isoquinoline alkaloids, only the Aristolactams subclass was tested for inhibition of DYRK1A and CDK1/cyclin B, associated with neurodegenerative diseases. Terpenes were the class of substances with the most reported biological activity, with monoterpenes standing out, which have 10 related biological activities. Anti‐inflammatory, antibacterial, antifungal, and anti‐trypanosome actions are some of the biological activities evaluated. Sesquiterpenes stood out in the evaluation of antitumor activity for HL‐60 cell lines, associated with leukemia. Acaricidal activity was also evaluated. Diterpenes and triterpenoids were tested for *Candida* spp.; anti‐inflammatory and insecticidal activity, respectively. These data, which are available in Table 2, allow us to note the relationship between the classes of chemical substances and the biological activities of the genus *Oxandra*.

In addition to the evaluation by chemical class, a critical discussion was also conducted on each species individually, including the evaluation of the secondary metabolites obtained, the particularities of each biological assay, and general comments on the possibilities and challenges related to bioprospecting of the species.

*O. xylopioides*, being the most phytochemically studied species, boasts over 20 compounds from various extracts, with unique alkaloids such as oxoaporphine derivatives and monoterpenes like isoespintanol [6, 7, 8, 9, 10, 11, 12, 43, 44, 45]. Most notably, its biological profile is the most diverse among the species reviewed: for example, isoespintanol shines with antioxidant activity higher than commercial standards, outperforming both BHT and thymol in FRAP and DPPH methods [14, 15], aside from potent antimicrobial activities, including antibiofilm action that outperforms ciprofloxacin and AFB against pathogens such as *P. aeruginosa* and *Candida* spp. [21, 22, 23, 24, 25, 26]. Considering that this plant is traditionally used to treat fever, it was coherent to evaluate biological activities related to infections and inflammations [5]. The main drawbacks relate to the high dependence on in vitro studies, which greatly limits its translational capability. In this regard, future studies should be addressed to in vivo models to confirm its anti‐inflammatory [8, 13, 18, 19, 20, 27, 28], cardioprotective, and antidiabetic properties that could place it as a polyvalent candidate for pharmacological development.

*O. espintana* has a more limited phytochemical scope, centered on aromatic monoterpenes like espintanol and sesquiterpenes from essential oils [29, 30]. Even so, its biological activity indicates applications. Espintanol has antiparasitic activity against Leishmania and Trypanosoma, with IC_90_ values in the range of 10–50 µg/mL, comparable to reference drugs [29], showing promise for neglected tropical diseases but limited so far only to in vitro data. The essential oil has acaricidal activity, too, with an LC_50_ of 13.24 µL/mL [30], besides an agrochemical aspect. Further investigation is necessary for comparison studies with other species of Annonaceae regarding environmental safety and effectiveness compared to synthetic pesticides.

*O. asbeckii* boasts a unique alkaloid profile, including the first aristolactams reported in this genus [31, 32, 33], and offers several interesting pharmacological and non‐traditional potentials. Velutinam is a potent kinase inhibitor, with IC_50_ values of 0.6 µM against DYRK1A and 1.5 µM against CDK1/cyclin B [33], which makes the species particularly promising against neurodegenerative disorders such as Alzheimer's disease, in which those targets contribute to tau pathology and neuronal dysfunction. This activity is unique among the ones reviewed here, which do not boast similar neurologic targets, but further mechanistic elucidation is still warranted. The crude alkaloid extract from this plant also exhibits anticorrosive action via a mixed‐type inhibition mechanism [34]. This may possibly inspire greener alternatives to protect metals from acid attack; in a junction between phytochemistry and materials science, this deserves further attention.

For *O. lanceolata* and *O. sessiliflora*, the focus is on sesquitterpene‐rich essential oils [35, 36, 38], being *O. sessiliflora* the one that offers more solid biological evidence. Its essential oil presents cytotoxic potential, whose IC_50_ is about 6 µg/mL against leukemia cells HL‐60 [36]. This species outperforms others in antiproliferative tests, while sesquiterpenes present antiparasitic activity towards *T. cruzi* with EC_50_ values ranging from 16 to 47 µM [39] with no mammalian cell cytotoxicity. It should be mentioned, however, that intra‐specific variability in oil composition [36] points out the importance of standardizing the extraction methodology. *O. lanceolata*, in turn, presents no biological studies despite reporting 90 identified compounds [35], which constitutes a severe gap. The prioritization of this species may come to reveal its hitherto unexplored bioactivities, judging from the structural similarities to bioactive congeners in closely related species. Considering the chemical composition of the essential oil of *O. espintana*, which includes the presence of the sesquiterpene espatulenol (**27**), a component that is also found in the essential oil of *O. lanceolata*, a possible application is acaricidal activity against females of *Tetranychus urticae*.

Finally, the alkaloid profile of *O. longipetala*, including oxoaporphines and azafluorenones [40, 41], completes the diversity within this genus. Its aqueous and ethyl acetate extracts exhibit potent antibacterial activity, presenting inhibition zones as large as 25 mm against *S. aureus* and *E. faecalis* [42], rivaling chloramphenicol. This suggests a possible route to develop antibiotics, but the lack of deeper mechanistic or *in vivo* data precludes broader comparisons. Among the different species, the bioactivity is mediated both by alkaloids and terpenes; however, the prevalence of preliminary in vitro studies requires interdisciplinary studies that include ethnopharmacology when appropriate to take full advantage of the potential of *Oxandra* as a source of new therapeutic and industrial compounds.

## 5 Conclusion

This review presents the first comprehensive assessment of the chemical and biological characteristics of *Oxandra* species, summarizing data from the six species investigated to date and reporting the identification of 64 compounds across several chemical classes, including flavonoids, alkaloids, terpenes, and steroids. Among these species, the leaves of *O. xylopioides* exhibited the broadest spectrum of biological activities, which included anti‐inflammatory, antioxidant, antibacterial, antifungal, and antidiabetic effects. Although only six of the 29 recognized species have been examined, the available evidence highlights the considerable potential of this genus as a source of bioactive molecules, exemplified by the alkaloid velutinam from *O. asbeckii*, which demonstrated promising inhibitory activity against DYRK1A and CDK1/cyclin B, key enzymes implicated in neurodegenerative disorders. Nevertheless, important knowledge gaps remain, particularly the absence of biosynthetic pathway elucidation, limited investigation of mechanisms of action, and the scarcity of in vivo studies capable of validating the pharmacological relevance of the identified compounds. Taken together, the findings reported to date suggest that the remaining, unexplored *Oxandra* species may also harbor structurally diverse and biologically significant metabolites. Expanding and integrating future research efforts will therefore be essential for uncovering new natural products of therapeutic interest and for deepening our understanding of the chemical diversity within *Oxandra* and, more broadly, American *Annonnaceae* species.

## Author Contributions

**Rayssa Cota Lopes**: wrote the original draft, created figures and tables for the article. **Francisco Paiva Machado**: wrote the original draft, resources wrote – review and editing. **Mateus de Freitas Brito**: organization of chemical structures, resources wrote – review and editing. **Thalisson Amorim de Souza**: investigation, validation, and wrote – review and editing. **Lucas Silva Abreu**: investigation, supervision, funding acquisition, resources, and wrote – review and editing. In addition, all authors have read and agreed to the published version of the manuscript.

## Conflicts of Interest

The authors declare no conflicts of interest.

## Data Availability

Data sharing is not applicable to this article as no new data were created or analyzed in this study.
